# Myalgic Encephalomyelitis or What? The International Consensus Criteria

**DOI:** 10.3390/diagnostics9010001

**Published:** 2018-12-20

**Authors:** Frank Twisk

**Affiliations:** ME-de-Patiënten Foundation, Zonnedauw 15, 1906 HB Limmen, The Netherlands

**Keywords:** myalgic encephalomyelitis, chronic fatigue syndrome, diagnosis, symptoms, muscles, neurology

## Abstract

Myalgic encephalomyelitis (ME) is a neuromuscular disease with two distinctive types of symptoms (muscle fatigability or prolonged muscle weakness after minor exertion and symptoms related to neurological disturbance, especially of sensory, cognitive, and autonomic functions) and variable involvement of other bodily systems. Chronic fatigue syndrome (CFS), introduced in 1988 and re-specified in 1994, is defined as (unexplained) chronic fatigue accompanied by at least four out of eight listed (ill-defined) symptoms. Although ME and CFS are two distinct clinical entities (with partial overlap), CFS overshadowed ME for decades. In 2011, a panel of experts recommended abandoning the label CFS and its definition and proposed a new definition of ME: the International Consensus Criteria for ME (ME-ICC). In addition to post-exertional neuroimmune exhaustion (PENE), a mandatory feature, a patient must experience at least three symptoms related to neurological impairments; at least three symptoms related to immune, gastro-intestinal, and genitourinary impairments; and at least one symptom related to energy production or transportation impairments to meet the diagnosis of ME-ICC. A comparison between the original definition of ME and the ME-ICC shows that there are some crucial differences between ME and ME-ICC. Muscle fatigability, or long-lasting post-exertional muscle weakness, is the hallmark feature of ME, while this symptom is facultative for the diagnosis under the ME-ICC. PENE, an abstract notion that is very different from post-exertional muscle weakness, is the hallmark feature of the ME-ICC but is not required for the diagnosis of ME. The diagnosis of ME requires only two type of symptoms (post-exertional muscle weakness and neurological dysfunction), but a patient has to experience at least eight symptoms to meet the diagnosis according to the ME-ICC. Autonomic, sensory, and cognitive dysfunction, mandatory for the diagnosis of ME, are not compulsory to meet the ME-ICC subcriteria for ‘neurological impairments’. In conclusion, the diagnostic criteria for ME and of the ME-ICC define two different patient groups. Thus, the definitions of ME and ME-ICC are not interchangeable.

## 1. Introduction

Myalgic encephalomyelitis (ME) is a distinctive neuromuscular disease [1,2], which was described in the medical literature between 1938 and 1993. Due to the introduction of chronic fatigue syndrome (CFS) [3,4] and the misconception that ME and CFS were ”similar disorders” [5], ME was rarely considered until 2011, when a group of experts proposed using the International Consensus Criteria to define ME (ME-ICC) in order to separate a distinct patient group from the heterogeneous group of patients with CFS [4]. This article reviews the similarities and differences between the original definition of ME [1,2] and ME-ICC [6].

### 1.1. ME (1938–1990)

ME has been described in the medical literature since 1938 [7], often due to outbreaks [8,9,10]. The endemic form of ME has been acknowledged since the 1950s [11,12]. ME, recognized as a clinical entity since 1956 [13], is primarily a neuromuscular disease, which is distinguished by muscle fatigability or prolonged muscle weakness after minor exertion; neurological symptoms indicating cognitive, autonomic, and sensory dysfunction; and a chronic relapsing course [1,2]. ME is often accompanied by various symptoms implicating the involvement of other systems, including the immune system, the gastrointestinal system, and the respiratory system [1,2].

### 1.2. CFS (1988–2018)

Much of the confusion relating to the diagnosis and the perception of ME originates from the introduction of the label CFS and its definition in 1988 [3]. The most commonly used definition of CFS, the Centers for Disease Control and Prevention (CDC) Fukuda definition, dates back to 1994 [4]. The only mandatory feature of CFS is (unexplained) chronic fatigue, which must be accompanied by at least four out of a list of eight ‘minor’ symptoms: substantial impairment in short-term memory or concentration; a sore throat; tender lymph nodes; muscle pain; multi-joint pain without swelling or redness; headaches of a new type, pattern, or severity; unrefreshing sleep; and post-exertional malaise (lasting for more than 24 h) [4]. Due to the disease’s nature, the case criteria for CFS [4] define a heterogeneous group of patients with chronic fatigue as the principle complaint.

Many researchers and clinicians consider ME and CFS to be “similar disorders” [14]. However, taking the definitions seriously, ME [1,2], a neuromuscular (polio-like) disease, and CFS [4], an ill-defined fatigue syndrome, are two different entities [15] with partial overlap. For this reason, ME [1,2] and CFS [4] simply cannot be replaced by the hybrid diagnosis systemic exertion intolerance disease (SEID) [5,16], as defined by the Institute of Medicine (now the National Academy of Medicine) [14].

### 1.3. ME-ICC (2011)

To separate a distinct patient group from the diffuse group of patients with CFS [4], a panel of international experts proposed the International Consensus Criteria for ME (ME-ICC) [6]. To meet the diagnosis of ME-ICC [6], a patient must experience post-exertional neuro-immune exhaustion (PENE), defined as “a pathological inability to produce sufficient energy on demand with prominent symptoms primarily in the neuro-immune regions”, as well as neurological impairments (at least one symptom from three of four symptom categories), immune, gastro-intestinal, and genitourinary impairments (at least one symptom from three of five symptom categories), and energy production/transportation impairments (at least one of four symptoms).

## 2. ME vs. ME-ICC: Similarities and Differences 

### 2.1. Similarities

#### 2.1.1. ME Is a Neurological Disease

The original definitions of ME [1,2] emphasize the great importance of neurological abnormalities, especially of symptoms implicating cognitive, sensory, and autonomic dysfunction as distinctive features [17]. The ME-ICC criteria acknowledge the significance of neurological impairments in a separate category (Table 1: symptom category B).

#### 2.1.2. ME Is a Multisystemic Disease

Both the original definition of ME [1,2] and ME-ICC [6] (Table 1) stipulate that ME is a multisystemic disease that can be associated with a wide range of symptoms, e.g., muscle pain, headaches, neurological dysfunction, immunological symptoms, gastro-intestinal complaints, as well as cardiovascular and respiratory symptoms. ME [1,2] and ME-ICC [6] both acknowledge the “variable involvement of cardiac and other systems” [1], alongside neurological dysfunction and musculoskeletal symptoms.

#### 2.1.3. ME Is Not a Psychogenic Disorder

The authors of the original definition [1,2] make it clear that, although biological abnormalities have not yet been demonstrated, ME should not be misinterpreted as a psychogenic illness. The ME-ICC [6] specifically exclude primary psychiatric and somatoform disorders, i.e., “mental disorders which manifest as physical symptoms”.

#### 2.1.4. ME Is Assumed to Be Associated with Neuropathology

The original definition of ME [1,2] proposes that ME is associated with inflammation of the brain and the spinal cord, while the ME-ICC [6] presupposes “neuropathology”.

Whether or not neuro-inflammation [19] is present in all ME [1,2] and ME-ICC [6] patients, and whether neuro-inflammation is causing all symptoms is yet unclear. However, this discussion of the definition of the disease, whether it be the original definition [1,2] or the ME-ICC [6], is independent of the most appropriate label for the disease.

### 2.2. Differences

#### 2.2.1. Muscle Fatigability or Prolonged Post-Exertional Muscle Weakness, Mandatory for the Diagnosis of ME, Is an Optional Element for the Diagnosis of ME-ICC

According to the original definition of ME [1,2], “Muscle fatigability is the dominant and most persistent feature of the disease and [...] a diagnosis should not be made without it. Restoration of muscle power after exertion can take three to five days or even longer” [20]. Muscle weakness is mentioned as just one of the examples of symptoms related to motor dysfunction (Table 1, symptoms type B4b) in the ICC, and motor weakness is not obligatory for the diagnosis ME-ICC [6]. Thus, muscle weakness, especially prolonged post-exertional muscle weakness, which is mandatory for the diagnosis of ME [1,2], is not required in order to meet the diagnosis of ME-ICC [6]. In more general terms, while the original definition [1,2] depicts ME as a neuromuscular disease, muscular symptoms are not required to meet the diagnosis of ME-ICC [6].

#### 2.2.2. Post-Exertional Neuro-Immune Exhaustion, Mandatory for ME-ICC, Is Not a Mandatory Feature of ME

Although the risks of over-exertion are acknowledged in the original descriptions of ME [1,2,20], the abstract concepts of post-exertional malaise [4] and post-exertional neuro-immune exhaustion [6] were never described as a mandatory symptom of ME [1,2,20]. To meet the original criteria of ME, [1,2,20] only two symptom clusters are mandatory: muscle fatigability or prolonged post-exertional muscle weakness and neurological disturbance, especially of cognitive, autonomic, and sensory functions, in addition to a prolonged relapsing course and variability of the symptoms [17].

#### 2.2.3. The Diagnosis of ME-ICC Requires Many More Symptoms Than the Diagnosis of ME

Although the original definition of ME [1,2] acknowledges the multisystemic nature of the illness, only two features are mandatory—post-exertional muscle weakness and neurological dysfunction. According to this original definition [1,2], cardiac and other bodily systems are variably involved in ME. ME-ICC [6] requires at least eight symptoms. In addition to post-exertional neuro-immune exhaustion, a patient must experience at least three neurological symptoms (Table 1, symptom category B), at least three symptoms related to immune, gastro-intestinal/genitourinary impairments (symptom category C), and one symptom associated with energy production or transportation impairments (symptom category D). Thus, the diagnosis of ME-ICC [6] requires the presence of symptoms that are optional for the diagnosis of ME [1,2].

#### 2.2.4. Autonomic, Sensory, and Cognitive Dysfunction (Mandatory for the Diagnosis of ME) Are Not Compulsory to Meet the ME-ICC Requirements for Neurological Impairments

One could argue that requiring at least three neurological symptoms (Table 1, category B) is sufficient to guarantee a neurological disturbance, mandatory for the diagnosis of ME [1,2]. However, according to the criteria for ME-ICC [6], a patient experiencing headaches (category B2a), unrefreshing sleep (category B3b), and muscle weakness (even without exertion) (category B4b) meets the requirements of symptom category B without experiencing any specific autonomic, sensory, and cognitive symptoms.

## 3. Summary

**Figure 1:**
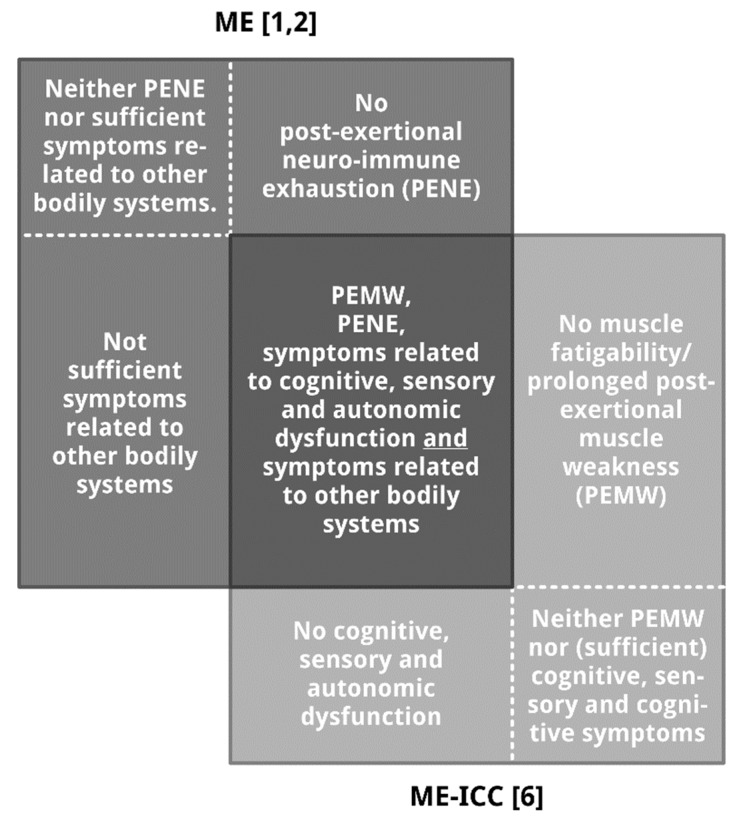
Overlap and differences between the case criteria of ME [1,2] and ME-ICC [6]. Note: Surface size does not reflect proportion. The (relative) number of patients in the seven subpopulations, especially the number of patients meeting the diagnosis of ME [1,2] and ME-ICC [6], and the patient subpopulation in the center are yet unknown. Figure 1 illustrates seven relevant patient subpopulations: patients meeting the diagnostic criteria of both ME and ME-ICC (the darkest grey rectangle in the centre), three ME patients subgroups not meeting the diagnosis ME-ICC (dark grey rectangles), and three groups of patients fulfilling the diagnostic criteria of ME-ICC, but not meeting the diagnosis ME (light grey rectangles).

The relationship between ME [1,2] and ME-ICC [6] is illustrated in Figure 1.

## 4. Discussion

ME [1,2] is a neuromuscular disease with distinctive features. The CFS case criteria [4] define a heterogeneous group of patients [21] with chronic fatigue as a common factor. Although a part of the CFS [4] patient group meets the diagnosis of ME [1,2] and a subgroup of patients with ME [1,2] qualifies as having CFS [4], the case criteria of ME [1,2] and CFS [4] define two distinct clinical entities [5]. The ME-ICC [6] criteria are meant to separate a specific patient group from the heterogeneous group of CFS patients [4]. This article reviews similarities and differences between the original definition of ME [1,2] and the ME-ICC [6], in order to determine objectively if the new definition of ‘ME’ (ME-ICC) [6] is a good alternative for the original definition of ME [1,2].

One could argue that the original definition of ME [1,2] should also be compared with the Canadian Consensus Criteria (CCC) for ME and CFS [22]. However, since the ICC [6] are meant to replace the CCC, as it was stated that “The Canadian Consensus Criteria were used as a starting point, but significant changes were made.” [6], a comparison between the original definition of ME [1,2] and ME- and CFS-CCC [22] can be considered irrelevant.

ME [1,2] and CFS [4] are two different clinical entities [15] with partial overlap. For this reason, ME [1,2] and CFS [4] cannot be replaced by the hybrid diagnosis SEID (ME/CFS) [5,16]. Hence, SEID is not a relevant alternative for either ME [1,2] or CFS [4]. This review shows that the case criteria for ME [1,2] and ME-ICC [6] also define two different patient groups. To unravel the etiology and pathophysiology of ME [1,2], ME-ICC [6], and CFS [4] and to develop effective treatments, it is crucial to diagnose patients accurately, using objective tests if possible, [23] and to stratify patients by duration of illness [24], age, and gender [25] in future research. 

## 5. Conclusions

Although the ME-ICC criteria [6] have relevant similarities with the original definition of ME [1,2], there are also several crucial differences. Muscle fatigability or long-lasting post-exertional muscle weakness after exertion, the distinctive feature of ME [1,2], is facultative for the diagnosis ME-ICC [6]. On the other hand, PENE, an abstract notion different from post-exertional muscle weakness, is the hallmark feature of ME-ICC [6] but is not obligatory for the diagnosis of ME [1,2]. While the diagnosis of ME [1,2] requires two types of symptoms (muscle fatigability or post-exertional muscle weakness and neurological dysfunction) [17], a patient has to report at least eight (one mandatory and seven variable) symptoms to meet the diagnosis of ME-ICC [6]. Autonomic, sensory, and cognitive dysfunction, mandatory for the diagnosis of ME [1,2], are not compulsory to meet the ME-ICC [6] requirements for neurological impairments. Although the symptoms required to meet the ICC-criteria for immune, gastro-intestinal, and genitourinary impairments (category C) and energy production and transportation impairments (category D) are often experienced by patients with ME [1], they are not compulsory for the diagnosis of ME according to its original criteria [1,2]. In summary, the diagnostic criteria for ME [1,2] and ME-ICC [6] define different patient groups and are not interchangeable. Future factor analysis studies [26] of the symptoms of patients meeting the discriminative definition of ME [1,2], which only requires two types of symptoms, should clarify to what extent ME [1,2] patients experience the mandatory and other symptoms required by the ME-ICC [6], how they meet the diagnosis of ME-ICC [6], and how many patients meeting the diagnosis of ME-ICC [6] comply with the original definition of ME [1,2].

## Figures and Tables

**Table 1:** The case criteria of myalgic encephalomyelitis (ME) [1,2] and the International Consensus Criteria for ME (ME-ICC) [6].

ME [1,2]	ME-ICC [6,18]
The pathognomonic features (of ME): 1. a complaint of general or local muscular fatigue following minimal exertion with prolonged recovery time ^a^; 2. neurological disturbance, especially of cognitive, autonomic, and sensory functions; 3. variable involvement of cardiac and other systems; 4. prolonged relapsing course. “Other characteristics include [...] variation in intensity of symptoms within and between episodes, tending to chronicity.” [1]	A. Post-exertional neuro-immune exhaustion (PENE): mandatory. B. Neurological impairments (at least one symptom from three of the four symptom categories): 1. Neurocognitive impairments a. Difficulty processing information: slowed thought, impaired concentration b. Short-term memory loss 2. Pain a. Headaches b. Significant pain in muscles, muscle-tendon junctions, joints, abdomen, or chest 3. Sleep disturbance a. Disturbed sleep patterns b. Unrefreshing sleep 4. Neurosensory, perceptual, and motor disturbances a. Neurosensory and perceptual symptoms b. Motor dysfunction C. Immune, gastro-intestinal, and genitourinary impairments (at least one symptom from three of five symptom categories): 1. Flu-like symptoms (recurrent or chronic, which typically activate or worsen with exertion) 2. Susceptibility to viral infections with prolonged recovery periods 3. Gastro-intestinal symptoms 4. Genitourinary symptoms 5. Sensitivities to food, medications, odors, or chemicals D. Energy production or transportation impairments (at least one of four symptoms): 1. Cardiovascular symptoms 2. Respiratory symptoms 3. Loss of thermostatic stability 4. Intolerance of extremes of temperature

^a^ “ME is a multisystem syndrome [...] distinguished by severe muscle fatigue following trivial exertion.” [1].
